# INPP5E and Coordination of Signaling Networks in Cilia

**DOI:** 10.3389/fmolb.2022.885592

**Published:** 2022-04-06

**Authors:** Renshuai Zhang, Jianming Tang, Tianliang Li, Jun Zhou, Wei Pan

**Affiliations:** ^1^ Key Laboratory of Animal Resistance Biology of Shandong Province, College of Life Sciences, Institute of Biomedical Sciences, Shandong Normal University, Jinan, China; ^2^ Zhenjiang Hospital of Traditional Chinese Medicine, Zhenjiang, China

**Keywords:** INPP5E, cilia, membrane-associated proteins, ciliopathies, signaling networks

## Abstract

Primary cilia are ubiquitous mechanosensory organelles that specifically coordinate a series of cellular signal transduction pathways to control cellular physiological processes during development and in tissue homeostasis. Defects in the function or structure of primary cilia have been shown to be associated with a large range of diseases called ciliopathies. Inositol polyphosphate-5-phosphatase E (INPP5E) is an inositol polyphosphate 5-phosphatase that is localized on the ciliary membrane by anchorage via its C-terminal prenyl moiety and hydrolyzes both phosphatidylinositol-4, 5-bisphosphate (PtdIns(4,5)P_2_) and PtdIns(3,4,5)P_3_, leading to changes in the phosphoinositide metabolism, thereby resulting in a specific phosphoinositide distribution and ensuring proper localization and trafficking of proteins in primary cilia. In addition, INPP5E also works synergistically with cilia membrane-related proteins by playing key roles in the development and maintenance homeostasis of cilia. The mutation of INPP5E will cause deficiency of primary cilia signaling transduction, ciliary instability and ciliopathies. Here, we present an overview of the role of INPP5E and its coordination of signaling networks in primary cilia.

## Introduction

The cilium is an antenna-like organelle that is ubiquitous in various cell types. They can be divided into two classes: motile cilia and non-motile cilia (also called primary cilia). Motile cilia have an axoneme that contains a central pair of microtubules surrounded by nine pairs of microtubules in a configuration called 9 + 2 and mainly distribute in the respiratory tract epithelium, ventricular ependymal epithelium, sperm and fallopian tube epithelium (Gudis and Cohen, 2010). However, primary cilia does not contain the central pair of microtubules and mainly distribute in the cone tube, vestibular sensory hair cells and olfactory epithelium (Takeda and Narita, 2012; Toriello and Parisi, 2009). The microtubule-based axoneme protruding from the basal body is enclosed by a bilayer lipid membrane (ciliary membrane) that is rich in membrane-associated proteins (Singla and Reiter, 2006). These proteins are pivotal in ciliary function and structure. Firstly, as a cell signal receiver and transmitter, cilia play essential roles in the reception and transmission of signals from extracellular stimuli. Signals are received through membrane proteins on the ciliary membrane and transmitted to downstream pathways, resulting in cascade reactions, such as Hedgehog (HH) and G-protein-coupled receptors (GPCR) pathway (Singla and Reiter, 2006). Moreover, cilia are unable to synthesize their own proteins and require intraciliary transport systems to transport these proteins. This process was conducted in a transition zone (TZ) which is maintained by the cilia membrane proteins. Within the TZ, the entry, localization of the transmembrane receptors and other proteins mediated formation and maintenance homeostasis of cilia are also elaborately regulated by membrane transport protein (Williams et al., 2011; Chih et al., 2012).

The localisation and activity of membrane associated proteins were dictated by phosphoinositides (PI). Due to distinct PI compositions, the protein composition of the ciliary membrane is different from that of the surrounding, contiguous plasma membrane. In additional, the distribution and abundance of PI were tightly modulated by the activity of PI kinases and PI phosphatases. Among these regulatory enzymes, INPP5E play critical roles in regulating the distribution and quantity of PI on cilia membrane. INPP5E is an inositol polyphosphate 5-phosphatase with a specific affinity for lipid substrates (Dyson et al., 2012). As a lipid signaling molecule, INPP5E regulates many cellular processes, including vesicle trafficking, cytoskeletal dynamics, protein synthesis, proliferation, and survival (Ooms et al., 2009). Here, we detailed summarize the roles of INPP5E in ciliary homeostasis and signal transduction.

## Cilia Associated Cellular Signalling

Cilia are ancient organelles with hair-like structures that extend from the cell body into the fluid surrounding the cell (Eley et al., 2005). Traditionally, motile cilia were thought to be a motor organ that generation of movement (Ran et al., 2021). In contrast, primary cilia serve an essential sensory purpose in transducing stimuli from extracellular environment to the cell interior to modulate the basic cellular processes (Singla and Reiter, 2006; Song and Zhou, 2020). These indicated that the main function of primary cilia is detection and transduction of cellular signalling. Among these pathways, Hedgehog (HH) and G-protein-coupled receptors (GPCR) pathway play critical roles in fulfiling the function of primary cilia (Ko, 2016; Loskutov et al., 2018).

The Hh pathway is a leading paradigm for ciliary signaling, and has diversity of functions in tissue homeostasis and proliferation (Briscoe and Thérond, 2013). It is initiated by Hh lipoprotein ligand binds to its transmembrane receptor protein patched (Ptch). Then, Ptch is inactivated and relieves smoothening (SMO), resulting in the activation of downstream targets through Gli transcription factors, which are processed from repressors to activators that organize the Hh transcriptional program (Bangs and Anderson, 2017; Zhang et al., 2021). GPCR signaling play critical roles in the sensory function of primary cilia (Schou et al., 2015). GPCRs are largest receptor superfamily in cilia which involve in numerous physiological functions. Once activated by heterotrimeric G proteins, the specific sites of GPCRs are phosphorylated by GRKs and recruit and bind with β-arrestins which sequencely activate downstream signal pathway, such as c-SRC and ERK1/2 (Eichel and von Zastrow, 2018).

### Ciliary Membrane-Associated Proteins and Ciliopathies

The composition of membrane-associated proteins confer the cilia with specific functions and structure. Due to lack of the ability to synthesis own proteins, the intracellular ciliogenesis pathway requires transportation, fusion and reorganization of ciliary proteins. And, membrane-associated proteins can modulate the structure and molecular composition of the cilia. Many studies have demonstrated that membrane-associated proteins, small Rabs, play critical roles in modulating ciliary structure. Currently, at least nine of the 66 Rabs have been reported to be involved in cilium formation and control of ciliary membrane protein levels (Hor and Goh, 2019). Rab8, which plays critical roles in polarized exocytosis in polarized epithelial cells and neurons, has been reported to promote extension of the ciliary membrane. Disruption of Rab8 function in zebrafish inhibited ciliogenesis. Another study demonstrated that Rab8 must coordinate with Rab11 to execute this function. Knockdown of Rab11 expression inhibited primary ciliogenesis (Knodler et al., 2010). ARL13B, highly enriched in cilia, stabilizes ciliary membrane integrity and anterograde IFT. Knock out this gene disrupts cilia architecture (Gigante et al., 2020). The mutation of ARL13B may cause Joubert Syndrome, a human disease now classified under the cluster of ciliopathies (Dilan et al., 2019). Furthermore, ciliary development and homeostasis are highly related to dynamic changes of ciliary membrane associated proteins. The BBS proteins were also involve in these process. They comprise a family of at least 11 proteins that localize to cilia and/or ciliary basal bodies (Blacque and Leroux, 2006; He and Axelrod, 2006). Evidence from studies in model organisms such as *C. elegans*, *Chlamydomonas*, *Xenopus laevis* and mice indicates that BBS proteins assist in the organization of intracellular trafficking and in coordinating motors responsible for anterograde IFT (Snow et al., 2004), as well as in recruiting PCP proteins to the ciliary basal body and cilium (Ross et al., 2005; Park et al., 2006). Mutution in these proteins are characterized by a series of disorders associated with ciliary dysfunction, such as obesity, pigmentary retinopathy, polydactyly, mental and growth retardation and renal failure (Mykytyn and Sheffield, 2004).

**FIGURE 1:**
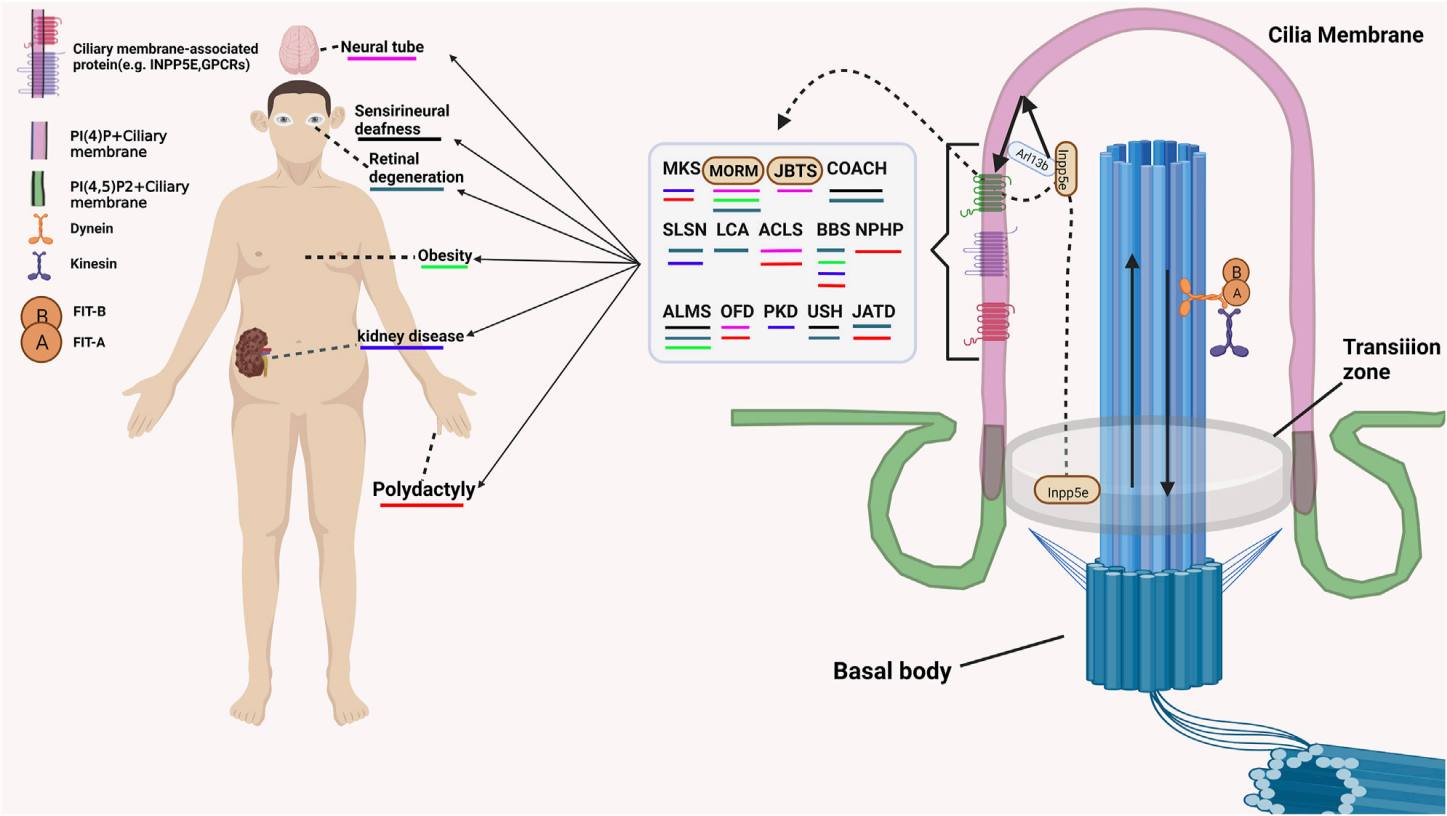
The example of cilia associated disease and related Symptom. MKS, Meckel-Gruber syndrome; MORM, Mental retardation, truncal obesity, retinal dystrophy, and micropenis; JBTS, Joubert syndrome; COACH, Cerebellar vermis hypo/aplasia, oligophrenia, congenital ataxia, ocular coloboma, and hepatic fibrosis; SLSN, Senior-Løken syndrome Arima syndrome; LCA, Leber congenital amaurosis; ACLS, Acrocallosal syndrome; BBS, Bardet-Biedl syndrome; NPHP, Nephronophthisis, truncal obesity, retinal dystrophy, and micropenis; ALMS, Alström Syndrome; OFD, Orofaciodigital syndromes; PKD, polycystic kidney disease; USH, Usher syndrome; JATD, Jeune asphyxiating thoracic dystrophy.

Except for modulating the structure and molecular composition of the cilia, many membrane-associated proteins also involve in receiving and transmitting extracellular signals. The G protein-coupled receptors (GPCRs), which are specifically located in the membrane compartment of the primary cilia, are involved in receiving various extracellular signals (Schou et al., 2015; Watabe et al., 2020). Multiple mutations of G protein-coupled receptors (GPCRs) cause functional disorders of cilia and lead to ciliary diseases. Some membrane-associated protein family not only participate in maintaining the structure and homeostasis of cilia, but also involve in regulating the cilia associated signalling. Rab23, one of small Rabs, inhibits Shh signaling by regulating Smoothened levels. However, the mechanism by which Rab23 modulates the expression of smoothened remains unknown elusive. The mutation of Rab23 in humans was characterized by carpenter’s syndrome (Hor and Goh, 2019). A recent study demonstrated that ARL13B is also a regulator of the Hh signaling pathway (Gigante et al., 2020). However, the regulatory mechanism of Hh signaling mediated by ARL13B was different from that of other ciliary genes that promote the Hh response and the production of Gli repressors and activators. Loss function of ARL13B may lead to an impaired response to Hh signaling and the production of activators but has no effect on the expression of the repressor Gli3 (Gigante et al., 2020). The mutation of ARL13B may cause Joubert Syndrome, a human disease now classified under the cluster of ciliopathies (Dilan et al., 2019). Arl6, also named as BBS3, is necessary for localization of the BBSome complex on cilia. Inhibition the expression of Arl6 cause reduction of ciliogenesis and Hh activity (Liu et al., 2016). Abnormalities in these functions of these proteins will cause various ciliary diseases (Pal et al., 2016; Long and Huang, 2019). The detaied ciliopathies and related symptom are shown in Figure 1.

### INPP5E Modulates Signaling Networks in Primary Cilia

**FIGURE 2:**
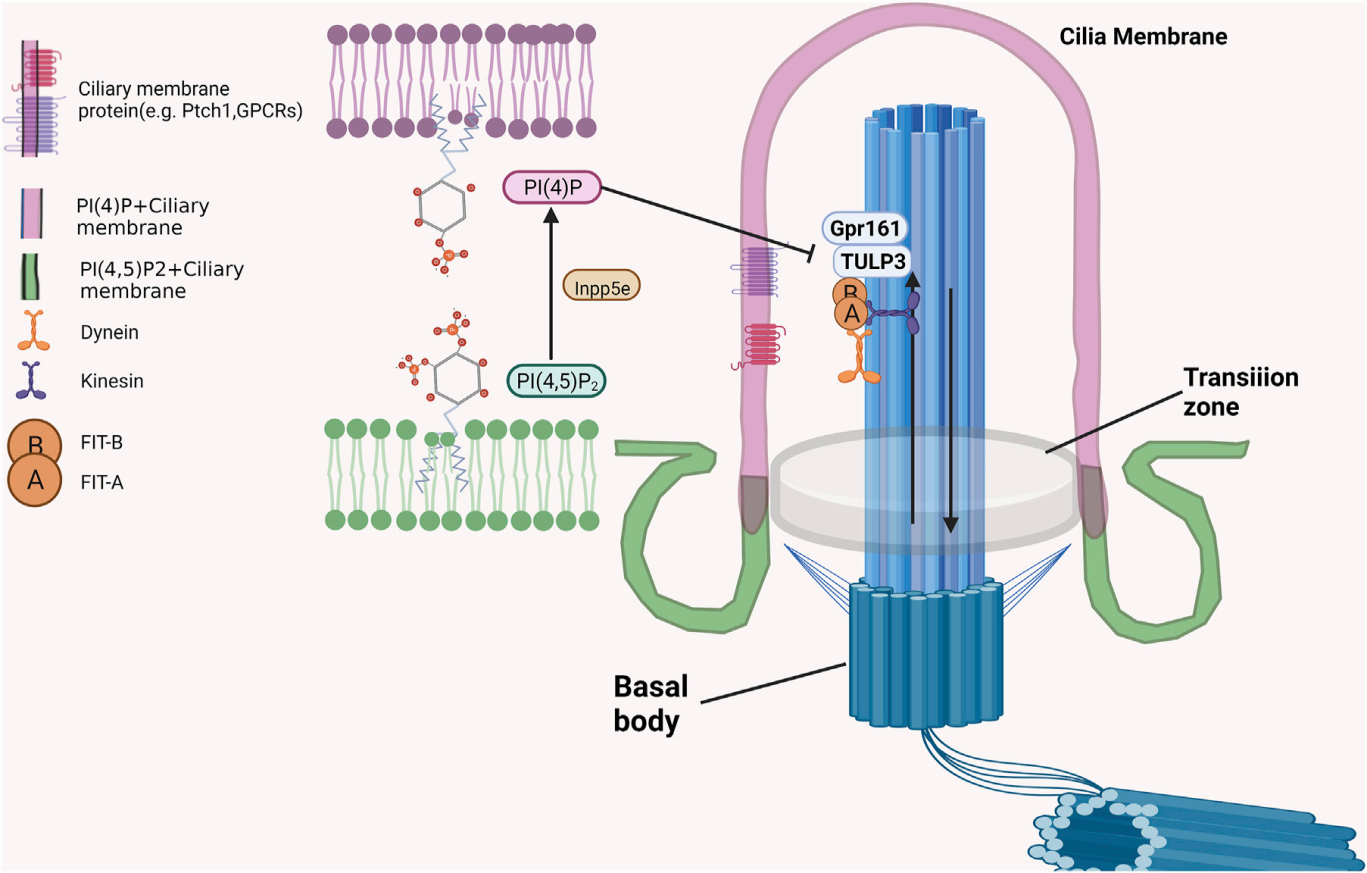
The critical roles of INPP5E in maintaining the PIs on cilia membrane.

As an inositol polyphosphate 5-phosphatase, INPP5E is mainly located in cilia in quiescent cells to maintain it is function and stability (Conduit et al., 2021; Kosling et al., 2018). A portion of INPP5E is also located in the lysosome, and its membrane anchoring and enzymatic activity are necessary for autophagy (Hasegawa et al., 2016; Sierra Potchanant et al., 2017). INPP5E located in the ciliary membrane could dephosphorylate phosphatidylinositol-4,5-bisphosphate (PtdIns(4, 5)P_2_) to generate phosphatidylinositol-4-phosphate (PtdIns(4)P) to maintain a PtdIns(4)P-high, PtdIns(4,5)P_2_-low environment, which was necessary for transmission of hedgehog signalling and blockage the entry of TULP3 and Gpr161 into cilia just as showed in Figure 2 (Chavez et al., 2015; Garcia-Gonzalo et al., 2015). After INPP5E inactivation, PI(4, 5)P_2_ accumulates at the apex of the ciliary body, while PtdIns (4)P is depleted. This process was accompanied by the recruitment of the PI (4, 5) P2-interacting proteins TULP3 and Gpr161 into cilia, and results in increased production of cAMP and repression of the Shh transcriptional gene Gli1, which affects the transmission of Shh signaling. (Han et al., 2019). Moreover, the ciliary needed a higher shh response to activate Smo when the function of INPP5E was lost. INPP5E regulates the shh response by adjusting the production of GliA/GliR in a time-dependent manner (Constable et al., 2020). By regulating SHH signaling, INPP5E could promote medulloblastoma progression through the PtdIns (3,4,5) P_3_/AKT/GSK3β signaling axis (Conduit et al., 2017). Other biological functions of cilia could also be regulated by the production or substrate of INPP5E. Recent studies have demonstrated that PIs in olfactory cilia participate in recognizing chemical odorants. The interplay (including relative abundance and localization) between phosphatidylinositol (3,4,5)-trisphosphate (PIP_3_) and phosphatidylinositol (4,5)-bisphosphate (PIP_2_), which are tightly regulated by INPP5E, play critical roles in these biological processes (Bielas et al., 2009). Furthermore, INPP5E regulate ciliary protein transport by controlling the interaction of the phosphoinositide component of the ciliary membrane with several centrosome proteins.

### INPP5E Functions Synergistically With Other Cilia Membrane-Associated Proteins

Although playing critical roles in biological processes of cilia, INPP5E may need to interact with membrane associated proteins to perform its function. On one hand, the ciliary membrane localization of INPP5E is determined by the membrane associated proteins. INPP5E, which lacks the sequence to which AR.L13B binds, was not detectable within cilia (Qiu et al., 2021). PDE6δ which is essential for the classification and entry of cilia of INPP5E also affect the retaining of INPP5E on the ciliary membrane (Fansa et al., 2016; Kosling et al., 2018). INPP5E targets primary cilia through a PDE6δ-dependent mechanisms. The mutation of PDE6δ, which loses the ability to bind with INPP5E, fails to target primary cilia (Thomas et al., 2014).

On the other hand, INPP5E could modulate the functions of membrane associated proteins in a direct or indirect manner. For example, the ability of Aurora kinase A (AURKA) in promoting the stability of cilia increases when binds with INPP5E. The transcription of AURK is also partly regulated by INPP5E which affect the activity of AKT (Plotnikova et al., 2015). INPP5E also plays critical roles in rod photoreceptor cells. Mutations in the RPGR gene are highly related to retinitis pigmentosa. Further investigation demonstrated that these mutations lost the ability to bind with INPP5E (Han et al., 2019; Zhang et al., 2019). Moreover, Tulp3, which localizes to primary cilia, is a negative regulatory factor in the Hh signaling pathway. The activation of Tulp3 was modulated by the substrate of INPP5E: PtdIns(4,5)P_2_, PtdIns(3,4)P_2_ and PtdIns(3,4,5)P_3_, which bind with the phosphoinositide binding domain of Tulp3 to promote MCHR1 trafficking to primary cilia (Mukhopadhyay et al., 2010). The product of INPP5E also participate in the initiation of ciliogenesis through modulate the function of ciliary membrane associated proteins. PtdIns(4)P, which is tightly regulated by INPP5E and PIPKIγ, could bind to TTBK2 and CEP164 which inhibits the localization of TTBK2 in M-centriole and the TTBK2-CEP164 interaction (Xu et al., 2016).

## Conclusion and Perspectives

Traditionally, motile cilia were thought to function by acting as mechanical sweepers. For example, motile cilia in brain ventricles promote the circulation of cerebrospinal fluid (Ringers et al., 2020); the debris and mucociliary of lungs and upper respiratory tract were cleared by cilia on epithelial surface of the respiratory tract (Legendre et al., 2021); and oviduct cilia transfer the fertilized egg to the uterus (Yuan et al., 2021). On the contrary, the primary cilium is a biosensor that transmits extracellular stimuli signals through ciliary membrane proteins to intracellularly. Recent investigations on the biology of cilia unveil many new functions and roles of both primary and motile cilia. Such as the role of motile cilia in organ homeostasis. And, primary cilia have been confirmed to be pivotal in tumorigenesis and chemosensation (Cao et al., 2021; Shi et al., 2019). These physiological processes are tightly modulated by ciliary membrane-associated proteins. To fulfill these special functions, the ciliary membrane need to have a diffferent composition of proteins that from that of the contiguous plasma membrane (Simons and Toomre, 2000). The quantity and localization of ciliary membrane-associated proteins are precisely regulated by the synergistic activity of PI kinases and PI phosphatases. Among these PI enzymes, INPP5E plays critical roles in ciliogenesis. An increasing number of findings demonstrate that INPP5E executes its function by interacting with membrane-associated proteins on cilia. However, the detailed mechanisms by which INPP5E and membrane-associated proteins cooperatively regulate the functions and structure of the cilium still remain elusive. It is necessary to elucidate these molecular mechanisms in the future investigations. Furthermore, more studies should focus on screening more membrane-associated proteins involved in regulating the function and structure of the cilium. A comprehensive understanding of how INPP5E and other membrane-associated proteins affect protein transport inside and outside the cilia and membrane protein structure as well as how they change the trend aggregation of second messengers may provide new insight for the diagnosis and treatment of ciliary diseases.
